# Cutaneous Larva Migrans Refractory to Therapy with Ivermectin: Case Report and Review of Implicated Zoonotic Pathogens, Epidemiology, Anthelmintic Drug Resistance and Therapy

**DOI:** 10.3390/tropicalmed10060163

**Published:** 2025-06-12

**Authors:** Bart J. Currie, Jessica Hoopes, Bonny Cumming

**Affiliations:** 1 Global and Tropical Health Division, Menzies School of Health Research, Charles Darwin University, Darwin, NT 0810, Australia; 2 Infectious Diseases Department, Royal Darwin Hospital, Darwin, NT 0810, Australia; 3 Animal Management in Rural and Remote Indigenous Communities, Darwin, NT 0820, Australia; jessica.hoopes@amrric.org (J.H.); bonny.cumming@amrric.org (B.C.)

**Keywords:** cutaneous larva migrans, zoonoses, One Health, ivermectin, albendazole, hookworms, antimicrobial resistance, Planetary Health

## Abstract

Cutaneous larva migrans (CLM) is attributed to zoonotic infection with animal hookworm larvae penetrating the human skin, usually the feet and legs. There is, however, a broad range of differential diagnoses, with the implicated hookworm species usually remaining speculative. Single-dose ivermectin is the most recommended current therapy, with repeat ivermectin doses sometimes required. With the massive global expansion of macrocytic lactone use in both livestock and companion animals, ivermectin resistance is being increasingly described in both helminths and ectoparasites. A case of CLM involving the foot of a healthy 37-year-old is described, with the failure of two doses of ivermectin 15 mg (240 μg/kg) a week apart. This occurred in the context of a remote work environment in tropical Australia with both companion animals (dogs and cats) and wildlife exposed to antiparasitic agents including ivermectin. A combination regimen of multiple doses of albendazole and ivermectin was curative. Parasites with multidrug resistance being described from animals now include hookworms in dogs which are resistant to pyrantel, benzimidazoles such as mebendazole and ivermectin. For relapsed CLM we now recommend a combination of ivermectin and albendazole therapy. This report supports the critical role for a One Health/Planetary Health approach to surveillance and response for emerging zoonoses and antimicrobial resistance in human and animal pathogens. This requires support for systematic approaches to foster and normalize communications and collaborations between human and animal health professionals, environmental scientists and ecologists and First Nations scientists who are the holders of Indigenous knowledge.

## 1. Introduction

Cutaneous larva migrans (CLM) is a clinical diagnosis to be considered in humans who develop one or more itchy, red skin lesions which migrate in a linear but often serpiginous fashion. It is not uncommonly reported in travelers returning from tropical and sub-tropical regions [1,2]. CLM is attributed to zoonotic infection with animal hookworm larvae penetrating the skin, most commonly on the feet, after direct contact with soil. The dog and cat hookworm *Ancylostoma braziliense* has historically been the main parasite implicated, but other animal hookworms are increasingly linked to CLM [3]. The differential diagnosis of CLM is broad and includes other migrating parasites, bacterial and fungal infections, insect-related skin diseases and linear skin inflammatory lesions following exposure to plants and jellyfish tentacles.

The treatment of CLM has evolved from various topical agents and cryotherapy to multiple doses of benzimidazoles, such as mebendazole and albendazole, and the current recommendation of single-dose oral ivermectin [4,5]. With increasing resistance globally to antiparasitic agents used for prophylaxis and therapy in animals and humans, CLM refractory to standard therapy seems a predictable future, and one that requires transdisciplinary One Health collaboration to address. Here, we present such a case and discuss the complexities of the epidemiology, zoonotic etiology and differential diagnosis, with eventual successful therapy using multidose combination therapy.

## 2. Patient Clinical History

A 37-year-old ecologist working in a remote First Nations community in Arnhem Land, Northern Territory, Australia, developed an intensely itchy rash on the dorsum of her right foot in March 2024. She described a “squiggly line of small red dots”, which “looked like a jellyfish sting across my toes”. Within 24 h, some blistering had developed, and the distal forefoot became painful and swollen, but the patient remained otherwise systemically well. There was no history of immunosuppressive disease or therapy and no recent use of anthelmintics. She sought medical attention three days later, and on examination, her right big toe had circumferential swelling and some blistering, with erythema spreading to her distal forefoot. She was prescribed antibiotics for a presumptive skin infection, which had progressed to cellulitis, with a spider or insect bite considered the possible primary event. Over a period of 10 days, the distal foot pain, inflammation and blisters improved, but the swelling persisted. Antibiotics received included cephazolin, amoxycillin/clavulanate and doxycycline. Blister swab microscopy showed no microorganisms, and cultures were negative for bacterial pathogens.

**Figure 1:**
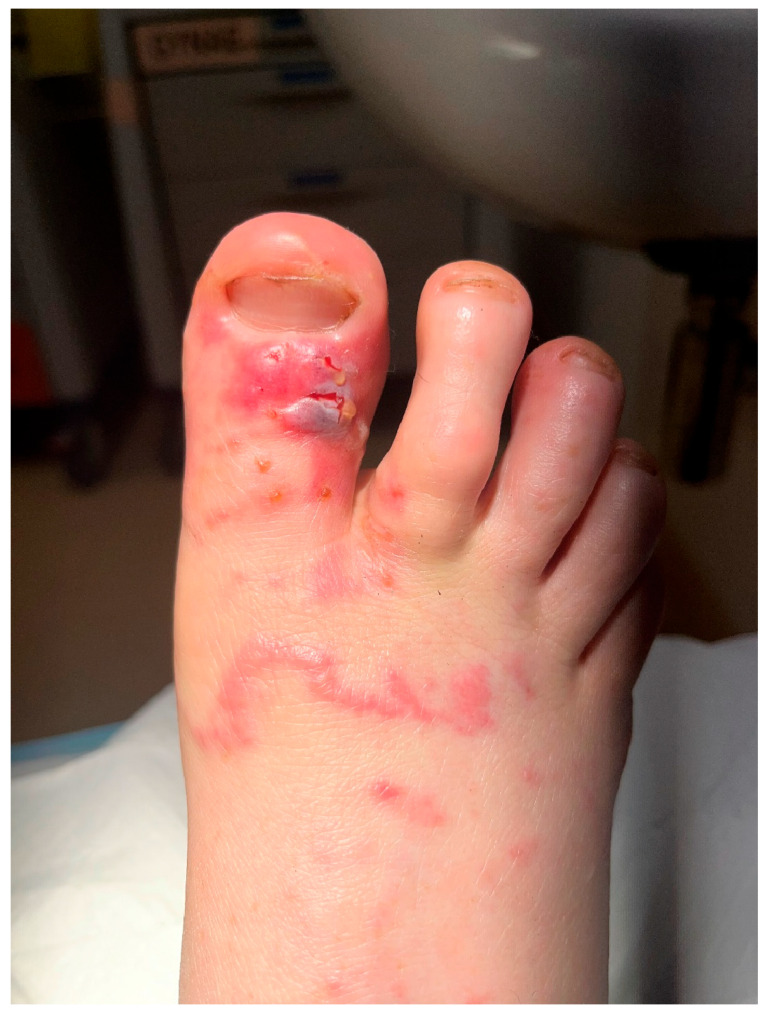
Right foot presumptive cutaneous larva migrans prior to treatment.

Two weeks after initial symptoms, she reported that new linear erythematous skin lesions were forming over the same area of her right forefoot, which were very itchy, and some had small blisters. On review of the images that she provided (Figure 1) and the epidemiology of her exposure in Arnhem Land, a presumptive diagnosis of CLM was made, and she was prescribed two doses of ivermectin 15 mg (240 μg/kg) a week apart, with fatty food. Blood tests showed an eosinophilia of 1.2 × 10^9^/L (NR 0.0–0.4) but a normal C-reactive protein of <0.3 mg/L (NR < 5.0), and *Strongyloides* spp. serology was negative.

**Figure 2:**
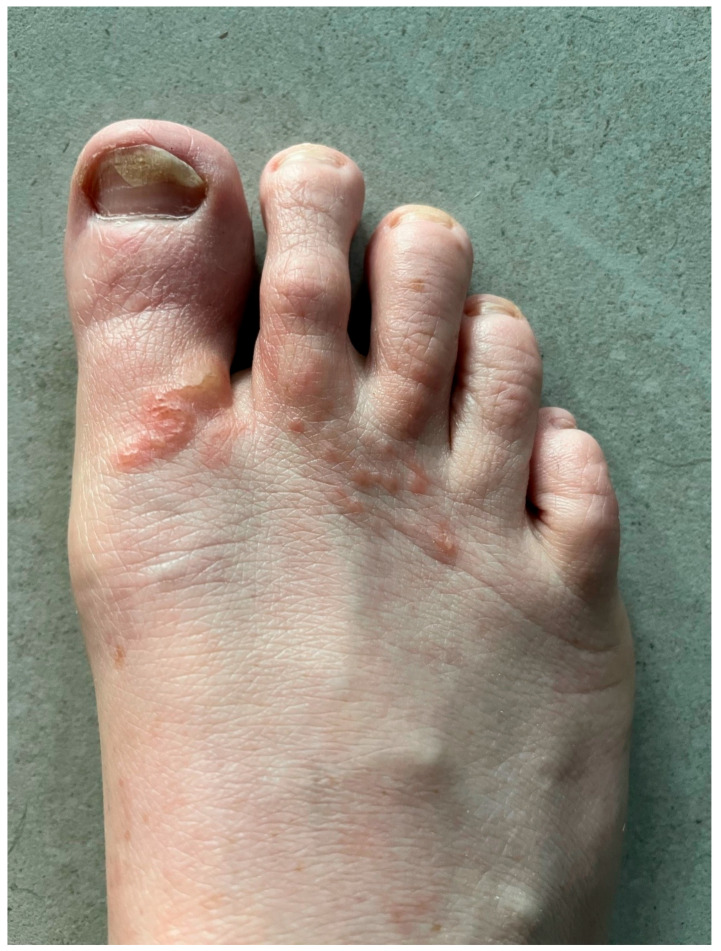
Right foot recrudescent skin lesions 2 weeks following therapy with ivermectin, 2 doses a week apart.

Having been told by her physician that “the ivermectin will stop it dead in its tracks–literally”, the patient was surprised to find that, after an initial improvement in swelling and itch, there was a progressive return of intensely itchy lesions on her right forefoot which would come and go, with some small blisters and “a new squiggly line” (Figure 2). Some of the itchy lesions on her forefoot were more proximal to those seen during the initial episode. Despite progression of the skin lesions, she remained systemically well. Repeat blood tests around 2 weeks after the second dose of ivermectin showed persisting eosinophilia of 1.1 × 10^9^/L but again a C-reactive protein of <0.3 mg/L and negative *Strongyloides* spp. serology. IgE was mildly elevated at 69 kIU/L (NR < 26). Given the treatment failure, despite the initial therapy including a repeat of the standard single-dose ivermectin therapy for CLM, she was treated with a combination therapy of albendazole 400 mg daily for 3 days and ivermectin 15 mg daily for 2 days, both with fatty food, and this was repeated after one week. Subsequent to this combination therapy, there was a rapid and complete resolution of her skin symptoms and signs, with no recurrence over the next 12 months.

The patient reviewed the manuscript and gave written informed consent for publishing her story and clinical photographs.

## 3. Exposure History

The location where the ecologist works encompasses remote homeland communities in tropical northern Australia, with stone and gorge country together with eucalyptus savannah. The soil is sandy, and there are abundant creeks and waterholes. Owned, free-roaming cat and dog populations are common in local communities, and feral cat populations have also been observed in the region. Native fauna include dingoes and many species of marsupials, birds, reptiles and amphibians. Prior to the development of skin lesions, she reported heavy rainfall in the area and abundant wet sand in her boots and living area. On later reflection, the patient recalled that, prior to the appearance of the rash and atypically for her, she had walked barefoot through a shallow puddle nearby her accommodation, and she felt that this was the likely infecting event.

## 4. History of Animal Management Programs in the Region Where Infection Occurred

Dogs are a longstanding and salient feature in many remote Australian communities, serving as companions, protectors and integral members of family and culture [6]. Despite their importance, limited access to regular veterinary services has resulted in overpopulation, poor health status and higher rates of infectious disease in dogs living in many remote areas [7]. As members of the community, companion animals receive considerable autonomy and are allowed to roam freely [8], which can increase their risk of disease exposure and the potential for zoonotic disease transmission [9,10]. These concerns have prompted the implementation of animal health and management programs in many remote communities, focusing on reproductive control and parasite prevention [11]. While these programs have historically focused on dogs, growing domestic cat populations have prompted the expansion of these programs to include cats in many areas [12]. In this instance, animal health and management programs have been delivered annually to all dogs and cats present in the community over the past five years. These programs have focused primarily on surgical desexing for population control and the provision of antiparasitic agents. These included ivermectin, the most widely used treatment in dogs, or the combination of isoxazolines such as afoxolaner and fluralaner, and broad-spectrum dewormers. Cats were exclusively treated with selamectin.

Laboratory determination of parasite prevalence in community animal health programs is challenging due to limited staffing and resources for diagnostic testing and the impracticality of routine surveillance, given the short duration and intermittent nature of veterinary service delivery in this context. As a result, empirical treatment is often based on clinical presentation and visual observation of endo- and ectoparasites to guide treatment selection, with broad-spectrum antiparasitic agents that balance effectiveness, ease of administration and cost being the most widely used. While there have been no published studies specifically evaluating the prevalence of endoparasites in the exposure area for the patient described in this report, studies over 3 decades assessing the prevalence of endoparasites in Australian remote community dogs have identified the presence of a number of zoonotic soil-transmitted helminths, including those responsible for the development of CLM [13,14,15,16,17,18,19,20,21,22,23]. However, studies on the prevalence of feline parasites in remote communities are much more limited [15,16,24], with *Ancyclostoma tubaeforme* being the only hookworm identified, although *A. caninum*, *A. braziliense* and *Unicinaria stenocephala*, have also been reported elsewhere in Australian cats [17,25,26]. Despite evidence of soil-transmitted helminths in Australia’s domestic dog and cat populations, the role of cats and dogs in zoonotic disease transmission is understudied.

Community dog health programs across Australia have historically relied on empirical single-dose regimens of high-dose, off-label ivermectin due to its low cost, ease of use and broad spectrum of activity against canine scabies and a number of zoonotic gastrointestinal helminths [27]. Compared to ivermectin products for dogs that have been approved for use by the Australian Pesticides and Veterinary Medicines Authority (AVPMA), the off-label doses required and used to improve ivermectin efficacy as a miticidal and endoparasitic agent are roughly 25–100 times the approved label doses used for heartworm prevention [28]. Treatments are often administered at the population level as part of veterinary service provision, under a similar premise to mass drug administration (MDA) programs in human medicine, with limited access to animal health products and veterinary services between programs.

## 5. Parasites That Could Be Potentially Causing the Cutaneous Larva Migrans and Other Differential Diagnoses

While CLM is classically attributed to the dog and cat hookworm *A. braziliense*, several other animal soil-transmitted roundworms (nematodes) have the potential to cause zoonotic larval migrans in humans. Most notable are the other dog and cat hookworms *A. ceylanicum*, *A. caninum* and *U. stenocephala* [3,29]. These zoonotic hookworms have larvae which generate an intense cutaneous inflammatory response on the penetration of human skin. This is in stark contrast to the dampened human immune responses to the co-evolutionarily evolved anthroponotic hookworms *A. duodenale* and *Necator americanus*, which result in no or minimal (“ground itch”; short-lived erythematous spots) immune reaction to the skin-penetrating larvae, allowing subsequent venous and lymphatic spread of larvae to the lungs. Invasion of alveoli follows, then migration to the trachea from where larvae are swallowed to then complete the development of patent human intestinal infection with adult worms and egg production.

There is a spectrum of human immune and clinical responses to the various zoonotic hookworms. *A. braziliense* engenders the most intense cutaneous response with the classical larva migrans and “creeping eruption”, while the responses to *A. ceylanicum*, *A. caninum* and *U. stenocephala* larvae are less severe and usually spontaneously resolve in a few days [29]. It is suggested that these latter hookworms are more likely to cause skin reactions in individuals sensitized by previous hookworm exposure [3]. This appears to contrast with more severe classical CLM attributed to *A. braziliense*, which is often described in residents from more affluent circumstances (with limited or no exposure to hookworms) who are visiting regions with large numbers of dogs and/or cats living in crowded circumstances. Other differences are that while *A. braziliense* never or extremely rarely results in patent human intestinal infections, *A. caninum* infections in humans are classically linked to an eosinophilic enteritis syndrome [30,31,32], and *A. ceylanicum* can behave very much like the anthropophilic hookworms, resulting in patent intestinal infection and even subsequent anemia [33,34,35,36,37,38]. Indeed, it has more recently been reported that *A. ceylanicum* is now the second most common hookworm infecting humans in the Asia Pacific region, with *N. americanus* the commonest [38].

*Strongyloides stercoralis* is a major human soil-transmitted roundworm that can cause fatal disseminated infection in immune-compromised people, most notably those on high-dose corticosteroid therapy. While classical foot CLM is not seen with initial *S. stercoralis* larval skin penetration, the parasite is capable of a human autoinfection cycle (non-soil), with infective larvae leaving the gastrointestinal tract and penetrating the skin, usually on the buttocks, trunk and abdomen [39,40]. Such “larva currens” can mimic CLM, but larval migration is considerably faster for larva currens (seen over minutes) than for CLM. *S. stecoralis* has been reported in dogs, cats and non-human primates, with molecular studies demonstrating a shared genetic haplotype in dogs, cats and humans [41,42,43,44,45,46]. Nevertheless, the role of these animals in the zoonotic transmission of strongyloidiasis in comparison to the human–soil–human cycle remains poorly understood. In rare cases, *Strongyloides papillosus* in sheep, goats and cattle and *Strongyloides westeri* in horses have also been implicated in the development of CLM [47].

Another zoonotic roundworm whose larvae can cause migratory lesions is *Gnathostoma spinergum*, as potentially can other *Gnathostoma* species. Hosts of the adult worm include cats, dogs and other carnivores. Humans are infected usually through eating undercooked intermediate hosts such as freshwater fish, shellfish, frogs and snakes [48]. Larvae do not reach maturity in humans, but episodic subcutaneous migrations of larvae can occur over years and cause itchy, swollen erythematous skin lesions, usually on the trunk and proximal limbs, unlike classical CLM, but occasionally on the feet. Human cases of gnathostomiasis have been rarely documented in Australia [49,50]. Other parasite infections listed to potentially cause migratory skin lesions are dracunculiasis (Guinea worm), fascioliasis, loiasis and paragonimiasis, which are not endemic to Australia, and sparganosis, which is endemic to Australian dogs, cats and wildlife but rarely documented.

Although not a helminth infestation, various insects can be hematophagous parasites of humans and animals. This includes sand fleas, also known as jiggers or chiggers, with tungiasis being a common zoonotic ectoparasitosis in the Americas and sub-Saharan Africa. Tungiasis is predominantly caused by *Tunga penetrans* and is seen occasionally in travelers after entry to Australia and other non-endemic countries. In tungiasis, the adult female flea penetrates the animal or human epidermis, commonly the feet, with the abdomen protruding through an open skin surface to allow mating, with subsequent egg production and deposition into sandy soil to complete the flea life cycle. The skin lesions are intensely itchy and may resemble early CLM, but larval migration is not seen.

Myiasis is the infestation of animals and humans with fly eggs and larvae (maggots), which can be deposited in skin wounds resulting in cutaneous myiasis with larvae feeding on the living tissue. Myiasis from the tumbu fly in Africa and the botfly in the Americas is occasionally found in travelers after entry to Australia and other non-endemic countries. Larvae from some fly species can cause “creeping myiasis”, with tunnels in the epidermis which can resemble CLM but thesespread more slowly and less extensively.

## 6. Wildlife and Cutaneous Larva Migrans

The animal reservoirs of the zoonotic hookworms and other pathogens responsible for CLM are likely to vary between different epidemiological circumstances, reflecting the complex global diversity of interactions between humans and both domesticated companion animals and wild canines, felids and other animals. Both semi-domesticated and wild animals were potential reservoirs for the CLM case reported here.

Research on the potential role of wildlife in the development of CLM is limited. However, wild canids (e.g., foxes and wolves), felids (e.g., panthers, lions, leopard cats and Bengal tigers), bears, hyaenas and civets have been suggested as potential reservoirs for CLM caused by *A. caninum* and/or *A. braziliense* [51]. In Australia, *A. ceylanicum* has been detected in dingoes and wild dogs [27,52], but zoonotic hookworm was not reported in red foxes [53]. Conversely, both *A. caninum* and *U. stenocephala* have been observed in red foxes in a study from Germany [54], suggesting foxes may still represent a potential reservoir for human infection. Globally, *G. hispidum* in wild boars and *Bunostomum phlebotomum* in cattle have been identified as possible causes of CLM [47,51]. A recent report from Ecuador implicated wild animals carrying an *Ancylostoma* sp. in a case of CLM [55].

## 7. Ivermectin and Other Anthelmintic Resistance in Australia and Globally

Anthelmintics have historically been developed and tested for the treatment of non-human parasitic nematodes that infect livestock and companion animals. There are few studies in the global literature on anthelmintic susceptibility for human hookworms [56,57,58], while the canine hookworm literature has historically focused mostly on pyrantel and benzimidazoles, including mebendazole and albendazole. Issues in anthelmintic susceptibility testing for hookworms include both the complexities in retrieving parasites for testing and the difficulties and lack of standardization of susceptibility testing, which includes both in vitro egg hatch and larval development and motility assays [57,59,60] and rodent and dog infection assays [61]. In addition, the speciation of animal hookworms can be problematic [26].

The first suspected case of pyrantel resistance in *A. caninum* was reported in 1987 following treatment failure in a greyhound puppy imported from Australia to New Zealand [62], with a clinical efficacy of 75.1% and 25.7% for pyrantel reported in subsequent clinical trials [63,64]. More recent studies have demonstrated widespread pyrantel resistance in canine hookworms in southeast Queensland [65] and an alarming widespread occurrence of benzimidazole resistance single-nucleotide polymorphisms in *A. caninum* from across Australia [66].

Parasite resistance to ivermectin and other macrocyclic lactones has been increasingly described globally, with emergence and selection linked to the widespread use of topical, oral and injectable products [67,68]. Intensive use of anthelmintics for the control of nematode infections in livestock has become particularly problematic, with multidrug resistance (MDR) described in Australia and many other countries [69,70]. In contrast to frequent MDR in cattle parasites, MDR in canine hookworms has developed much more slowly, with few cases reported until recently [61]. Nevertheless, with the increased use of prophylactic anthelmintics in pets and the expansion of mass drug administration (MDA) programs for soil-transmitted helminths and other parasites in humans, it seems likely that resistance to macrocyclic lactones in hookworms will become more common [58].

The first report of a naturally occurring strain of *A. caninum* being resistant to the macrocyclic lactone ivermectin as well as benzimidazoles involved a hookworm isolated in 2016 from a retired racing greyhound in the USA. The greyhound had a history of monthly heartworm preventative treatment [59]. It was noted that housing conditions and treatment regimens associated with racetracks can exert extreme selective pressure on small populations of hookworms, with potential for selection and fixation of resistant alleles. Subsequently, MDR in three further dogs infected with *A. caninum* was found, with what appears to be the same mutation associated with benzimidazole resistance [61]. Further reports of cases of MDR *A. caninum* have emerged with some spread from the greyhound population to the companion dog population [71,72,73]. While a number of mutations in the parasite β-tubulin gene have been shown to impart benzimidazole resistance in hookworms, and these mutations are also seen in benzimidazole resistance in multiple livestock nematodes [56], the mechanism of ivermectin resistance in *A. caninum* remains undetermined [59,73].

## 8. Discussion

For the patient with CLM reported here, the clinical presentation and history of exposure to sandy soil in a location with both cats and dogs are very much consistent with a diagnosis of CLM from a dog and/or cat hookworm. Differential diagnoses for the patient’s migratory skin lesions include the other zoonotic helminths and insects discussed above, erythema migrans (but Lyme borreliosis is not present in Australia), jellyfish stings (but well inland from the sea), allergic or direct irritant contact dermatitis (such as phytodermatitis and phytophotodermatitis from plants) and scabies and tinea (both clinically ruled out).

Assuming a diagnosis of CLM, what is concerning is that initial treatment with ivermectin was not curative, despite a regimen of two doses a week apart. Historically, CLM was treated with topical thiabendazole or cryotherapy, with oral benzimidazoles such as albendazole or oral ivermectin subsequently becoming standard therapy [4,5]. A small study in the early 1990s showed single-dose oral ivermectin to be superior to single-dose albendazole, with relapse common with albendazole [74]. Single-dose ivermectin subsequently became the most commonly recommended therapy for CLM, with high cure rates. Occasional failures or relapses have been noted, and these have responded to one or two supplementary doses of ivermectin [1,5,75].

There are a number of possible explanations for the failure of ivermectin in the case reported here. The ivermectin was a standard hospital prescription of commercial in-date tablets, each dose was above the 200 μg/kg recommended and was taken with food to maximize absorption [76]. The most likely scenario seems to be that the patient was infected with a hookworm that was resistant to ivermectin and that this resulted from hookworms in the local dog and cat population. A recent study of dogs in remote communities found a prevalence rate of 83.9% for *A. caninum* based on qPCR across three communities and fecal shedding in some dogs exceeding 10,000 (range 0–14,430) eggs per gram of feces [23], which increases the likelihood of environmental contamination and human exposure to hookworm larvae.

The intermittent nature of animal health programs and potential refugia in wild populations (e.g., feral cats, dingoes and foxes) differ from the selection pressures imposed by intensive parasite treatment programs in closed systems such as greyhound kennels, where resistance development has commonly been reported. However, like MDA programs in humans, population-level parasite treatment administration in cats and dogs may act as a potential driver for resistance development, and the movement of dogs between communities presents an additional opportunity for the introduction of resistant hookworms into the area. Additionally, the free-roaming nature of dogs in communities presents challenges for the management of animal wastes, and coprophagic behavior may increase the potential for exposure to subtherapeutic doses of ivermectin and other macrocyclic lactones that are excreted largely unchanged in animal feces [77]. Further, with pervasive sanitation challenges in many remote communities, free-roaming dogs may additionally be exposed to subtherapeutic doses of ivermectin through coprophagy of human feces [78]. A recent study utilizing off-label ivermectin for the treatment of *Ancyclostoma caninum* in dogs in remote communities demonstrated a 90% cure rate, with 90% of dogs achieving a 90% egg reduction rate 7–11 days after treatment [23]. While these results suggest a good overall efficacy for ivermectin against canine hookworm, they also highlight the need for the ongoing monitoring of community animal health programs to ensure sustained parasite control and effectively mitigate risks to human and animal health. However, this is often not possible in remote field-based settings, given the nature of service delivery in this context. These challenges also underscore the need for broader transdisciplinary One Health collaboration between the human, animal and environmental health care fields in remote and resource-limited environments in addressing health conditions at the human–animal–environmental interface.

Following the relapse of CLM in the patient, the cure was achieved by combination therapy with 3 consecutive days of albendazole and 2 consecutive days of ivermectin therapy, repeated after 1 week. We have been using combination therapy with ivermectin and albendazole for patients with asymptomatic eosinophilia, and the combination has advantages in broadening the helminth cover of the individual anthelmintics [79,80] as well as potential synergistic activity. A similar combination therapy approach with a fixed-dose coformulation of ivermectin and albendazole has also been suggested for the control of strongyloidiasis, potentially providing both increased therapeutic efficacy against the range of soil-transmitted helminths as well as mitigating against evolution of ivermectin resistance [81]. This fixed-dose coformulation of ivermectin–albendazole has recently been found to be superior to albendazole alone against *T. trichuris* and hookworms [82,83].

Given that the larger studies of ivermectin use for CLM have shown some, albeit still uncommon, failures of single-dose ivermectin [1,5,75] and similarly that 3 consecutive days of albendazole is usually but not always curative [84], our approach to CLM has been to repeat the ivermectin dosing after 1 week. This conservative approach clearly failed in the case reported here, which may represent the zoonotic transmission of an ivermectin-resistant dog/cat hookworm. Continued clinical surveillance seems prudent to document and report similar cases. Meanwhile, we recommend the consideration of combination ivermectin and albendazole therapy for relapsed CLM, as was successfully used for the case we report.

This case report and review shows the critical role of a One Health/Planetary Health approach to the surveillance and response for emerging zoonoses and the increasing scale of antimicrobial resistance in human and animal pathogens. What is required is support for systematic approaches to foster and normalize communications and collaborations between human and animal health professionals, environmental scientists and ecologists and First Nations scientists who are the holders of Indigenous knowledge.

## Data Availability

The original contributions presented in the study are included in the article, further inquiries can be directed to the corresponding author.
